# Novel Device for Intraoperative Quantitative Measurements of Extraocular Muscle Tensile Strength

**DOI:** 10.3390/bios15060347

**Published:** 2025-05-30

**Authors:** Hyun Jin Shin, Minung Park, Hyunkyoo Kang, Andrew G. Lee

**Affiliations:** 1 Department of Ophthalmology, Konkuk University School of Medicine, Chungju 27478, Republic of Korea; shineye@kuh.ac.kr; 2 Department of Ophthalmology, Konkuk University Medical Center, Seoul 05030, Republic of Korea; 3 Research Institute of Medical Science, Konkuk University, Seoul 05030, Republic of Korea; 4 Institute of Biomedical Science & Technology, Konkuk University, Seoul 05030, Republic of Korea; 5 Department of Mechatronics Engineering, Konkuk University Glocal Campus, 268 Chungwondae-ro, Chungju-si 27478, Republic of Korea; pmu0825@naver.com; 6 Department of Ophthalmology, Blanton Eye Institute, Houston Methodist Hospital, Houston, TX 77030, USA; aglee@houstonmethodist.org; 7 Department of Ophthalmology, Neurology, Neurosurgery, Weill Cornell Medicine, New York, NY 10065, USA; 8 Department of Ophthalmology, University of Texas Medical Branch, Galveston, TX 77555, USA; 9 Department of Ophthalmology, UT MD Anderson Cancer Center, Houston, TX 77030, USA; 10 Department of Ophthalmology, Texas A and M College of Medicine, College Station, TX 77807, USA; 11 Department of Ophthalmology, University of Iowa Hospitals and Clinics, Iowa City, IA 52242, USA; 12 Department of Ophthalmology, Baylor College of Medicine and the Center for Space Medicine, Houston, TX 77030, USA; 13 Department of Ophthalmology, University of Buffalo, Buffalo, NY 14214, USA

**Keywords:** strabismus, extraocular muscles, tensile strength, muscle tension, quantitative measurements

## Abstract

Understanding the tensile properties of extraocular muscles (EOMs) is crucial for successful strabismus surgery and accurate predictions of surgical outcomes. Assessments of EOM tensile strength are traditionally highly dependent on the expertise of the ophthalmic surgeon, since they involve manually pulling the EOM in opposite directions. This approach only provides subjective measurements that are not quantifiable. Previous quantitative approaches have utilized various devices such as implanted force transducers or dial tension gauges connected to muscle tendons with nylon sutures, but these methods are complex and so are rarely used outside of research settings. Consequently, the goal of this study was to create a quantitative and clinically applicable device for assessing EOM tensile strength. This developed device uses a strabismus hook connected to a strain gauge load cell that measures the tensile force and includes a tilting sensor to ensure that the hook is pulled at a consistent angle when a force is applied. The performance of the device was tested on 22 EOMs in 11 patients with intermittent exotropia during surgery for resecting the medial rectus (MR) and recessing the lateral rectus (LR) under general anesthesia. The measured tensile strengths of the MR and LR were 284.9 ± 58.3 and 278.3 ± 64.6 g (mean ± SD), respectively. In conclusion, the novel device developed in this study for quantitative measurements of EOM tensile strength in clinical settings will facilitate understanding of the pathophysiology of strabismus, as well as of the mechanical properties of the EOMs, and enhance the precision of surgical interventions.

## 1. Introduction

Strabismus is a condition where the foveas do not align properly when focusing on an object. This misalignment can cause the eyes to point in different directions, leading to double vision with an abnormal head posture or the brain suppressing the input from one eye to avoid double vision, which may result in reduced depth perception or even decreased vision in the suppressed eye [1,2]. Strabismus surgery aims to correct this misalignment by adjusting the positions of the extraocular muscles (EOMs). To achieve proper alignment, the procedure involves modifying the tension in individual EOMs by either resecting (shortening) or recessing (lengthening) them, which will strengthen or weaken them, respectively [3].

Evaluating the mechanical properties of EOMs is important for both the success of strabismus surgery and for a full understanding of the pathophysiology for the misalignment [4,5,6]. The tensile strength corresponds to the force required to stretch or pull a material until it deforms by a certain amount. In the case of an EOM, this strength determines how much force is required to stretch the muscle, and this characteristic is important in various surgical procedures. For example, during EOM resection surgery, the EOM is pulled in opposite directions using a strabismus hook. Even if a specific amount of muscle is planned to be resected, the actual amount removed may vary depending on the tensile strength of the muscle and the force applied by the surgeon; for example, a greater proportion of the muscle may be resected if it is stretched less [7,8]. Additionally, in restrictive forms of strabismus such as Graves’ ophthalmopathy, measurements of EOM tensile strength will help to estimate the degree of EOM fibrosis and the disease severity [9,10].

Recent research on EOMs includes several efforts to quantitatively analyze its biomechanical characteristics. A finite element model incorporating active EOM contraction and optic nerve mechanics has been developed to simulate and quantify medial rectus tension and related biomechanical interactions [11]. MRI-based volumetric analyses have also been conducted to assess the relationship between EOM volume and motility impairment [12], and the cross-sectional areas of the rectus muscles were evaluated in highly myopic subjects using high-resolution MRI [13]. Furthermore, to enhance the stability, accuracy, and efficiency of robotic systems involved in ocular procedures, load cell-based force and torque feedback mechanisms have been applied [14,15].

However, the current methods used to evaluate EOM tensile strength are highly dependent on the experience and skill of the ophthalmic surgeon as they pull the strabismus hooks in opposite directions to stretch the EOM. This method has the limitation that the tensile force exerted by the strabismus hook while stretching the EOM is assessed subjectively by the surgeon and, hence, can vary even in the same patient [16]. This results in inaccuracy and variability in the evaluation and measurement of EOM tensile strength, which can lead to overcorrection or undercorrection after strabismus surgery [17,18]. Reducing these errors requires precise and objective methods for measuring the EOM tension. Previous attempts to quantitatively analyze EOM tensile strength have employed various devices such as implanted force transducers [19] or dial tension gauges connected to the muscle tendon with nylon sutures [20]. However, these measurement approaches are highly complex and, therefore, are seldom used outside of research settings.

In this study, we developed a novel device for measuring the tensile strength of human EOMs that can be easily integrated into clinical practice. The proposed device features a strabismus hook connected to a strain gauge load cell to measure tensile forces, along with a tilting sensor to eliminate inaccuracies caused by hook misalignment. We believe that this quantitative measurement tool will allow clinicians to better understand the pathophysiology of strabismus and the mechanical properties of EOMs, thereby ultimately enhancing the precision of strabismus surgery.

## 2. Materials and Methods

### 2.1. Design of the EOM Tensile-Strength Measuring Device

**Figure 1:**
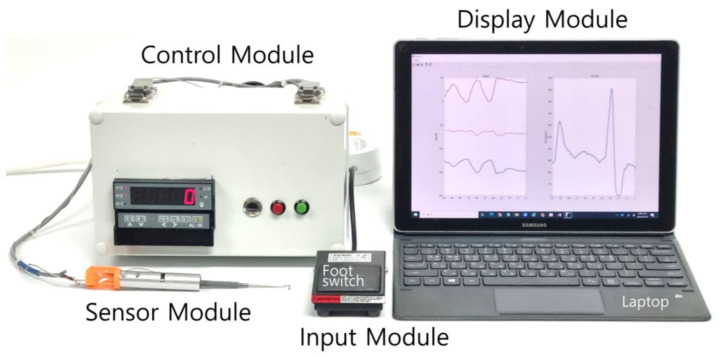
The novel extraocular muscle (EOM) tensile-strength measuring device (TSMD). The TSMD comprises a hook-type sensor module, a control module, a display module, and an input module.

**Figure 2:**
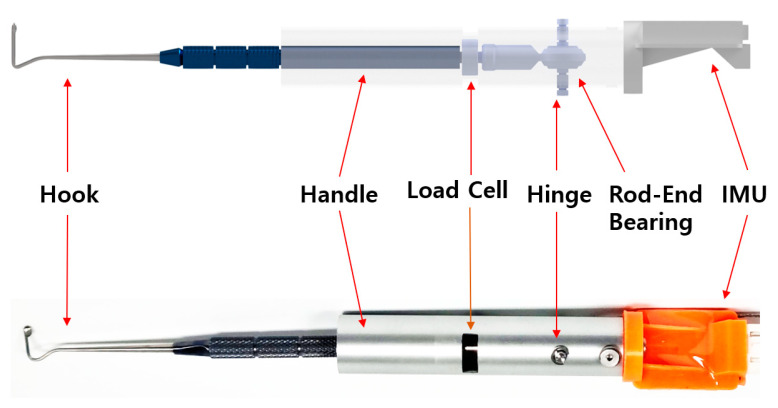
A schematic and photograph of the sensor module. A mechanical tap connects the hook to one end of the load cell, and a rod-end bearing is attached to the opposite end. The shaft of the rod-end bearing serves as a hinge. An inertial measurement unit (IMU) sensor is mounted on the top of the handle.

The tensile-strength measuring device (TSMD) for EOMs developed in this study comprises the following four modules (Figure 1): (i) a sensor module, (ii) a control module, (iii) a display module, and (iv) an input module. The sensor module includes a rod-end bearing (PHSCM3, MISUMI Korea, Seoul, Republic of Korea), a miniaturized compressive-tensile-type load cell (LCM100-10lb, Futek, Irvine, CA, USA), an MEMS (microelectromechanical system)-based inertial measurement unit (IMU) sensor (EBIMU-9DOFV4, E2BOX, Hanam, Gyeonggi-do, Republic of Korea), and a Jameson strabismus hook (Storz E0586, Karl Storz, Tuttlingen, Germany). The cylindrical load cell has threaded sections protruding from both ends, one of which is attached to the hook while the other one is connected to a rod-end bearing. The selected load cell has a natural frequency of 26 kHz. Based on the frequency response characteristics of second-order systems, the reliable measurement frequency range is normally limited to about one-tenth of the natural frequency to minimize phase and amplitude distortion. Accordingly, the usable frequency range is around 2.6 kHz, which is sufficient to evaluate EOM tension in transient states. The axis of the rod-end bearing acts as a hinge, allowing compensation for the tilting angle of the force applied to the end of the hook. This mechanical design allows the bearing to rotate and realign the direction of the pulling force to match the axis of the load cell, compensating for minor angular misalignment occurring. However, since the rod-end bearing has a limited mechanical swing range, we integrated a IMU-based tilt sensor that provides real-time orientation feedback to the operator during surgery. The tilt sensor does not correct the force directly but rather serves as a guidance tool to help the user to maintain proper sensor orientation and avoid significant misalignments. The operating range for the load cell and IMU sensor were set from 0 to 2.5 kg and from 0 to 360 degrees, respectively. Figure 2 shows a photograph of the sensor module assembly.

**Figure 3:**
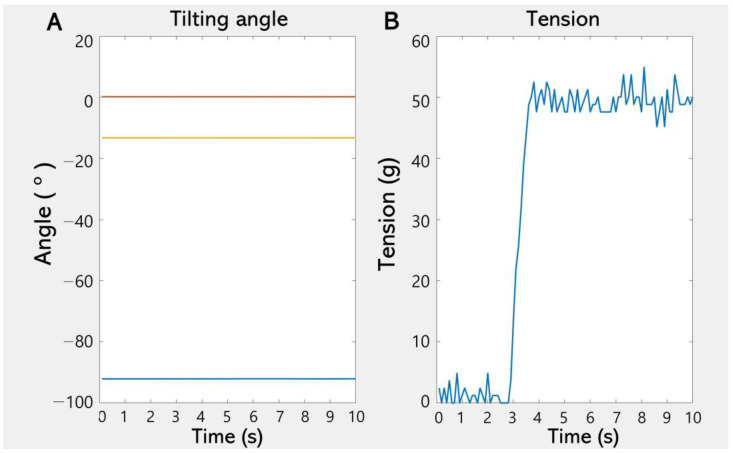
A screenshot of the display module. The tilting angle (**A**) and force (**B**) are displayed on separate graphs in real time.

**Figure 4:**
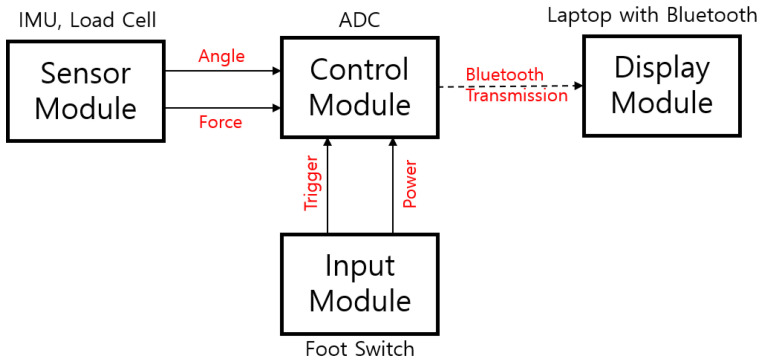
The signal flow of the TSMD. The control module collects the force and tilting-angle signals, which are sent wirelessly to the laptop via Bluetooth. The TSMD measurements are activated by a foot switch.

The control module consists of an amplifier (Al-50, Caskorea, Gyeonggi-do, Republic of Korea) for amplifying the signals from the load cell and an Arduino-compatible microcontroller board (Orange board, Kocoafab, Seoul, Republic of Korea) for serial communication and analog-to-digital conversion (ADC). Using an ADC sampling resolution of 10 bits produced a sensitivity threshold for measuring forces of 1.22 g. The sampling frequency of the ADC was 100 Hz. The measured tilting angle and force were wirelessly transmitted to a laptop (Galaxy Book, Samsung Electronics, Gyeonggi-do, Republic of Korea) via a Bluetooth module (HC-06, Guangzhou HC Information Technology, Guangzhou, China). The laptop displayed the tilting angle and force in real time and stored the data using mathematical computing software (MATLAB version R2021b, MathWorks, Natick, MA, USA), as shown in Figure 3. A schematic of TSMD signal flow is presented in Figure 4.

### 2.2. Calibration of the EOM TSMD

**Figure 5:**
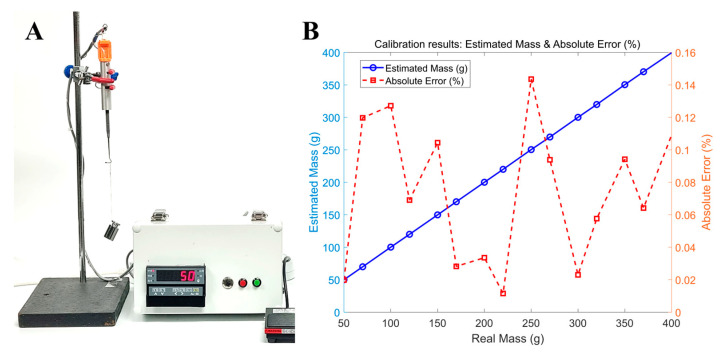
The calibration of the TSMD. (**A**) The calibration setup for the TSMD used to characterize the relationship between the applied mass and the measured output voltage. (**B**) Calibration results between the real mass and the estimated mass along with the absolute error.

The TSMD was calibrated in order to characterize the relationship between the output voltage and the mass attached to the hook of the sensor module. As shown in Figure 5A, the sensor module was gripped using a three-prong finger swivel clamp with rubber-coated jaws for increasing the grip. A test weight was attached to the hook of the sensor module via a wire, and the amplifier output was measured by the control module over a range from 50 to 400 g. The calibration process yielded the following equation with an R^2^ value of 99.99963%, confirming that this equation could be used to reliably estimate the actual weight over the specified measurement range of 50–400 g:

(1) y=253.47x+0.1872

where y is the estimated mass in grams and *x* is the measured amplifier voltage. The absolute error ranged from 0.1435% to 0.0115%, as shown in Figure 5B.

**Figure 6:**
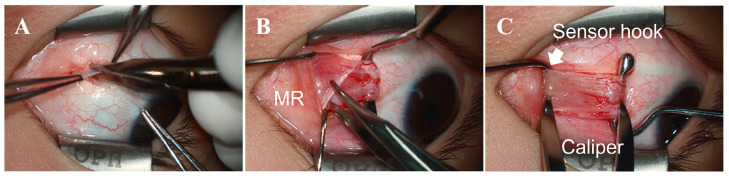
Photographs of the procedure for measuring EOM tensile strength using our novel device. (**A**) Approaching the EOM through a conjunctival incision. (**B**) Exposing the medial rectus muscle (MR). (**C**) Hooking the MR using a Jameson strabismus hook connected to the sensor module. The MR was secured with an opposing hook, and the two hooks were pulled 10 mm in opposite directions to measure the tensile strength. The IMU sensor detected the tilting angle to ensure accurate measurements.

To stretch an EOM, the ophthalmic surgeon gripped the handle of the sensor module and places the Jameson strabismus hook at the insertion point of the medial rectus (MR) and lateral rectus (LR). The opposing hook was then placed on the opposite side of the muscle insertion, positioned facing the hook of the sensor module, and the two devices were pulled in opposite directions. The tensile force pulling the EOM was then measured by the load cell attached in the middle of the Jameson strabismus hook and the rod-end bearing, as shown in Figure 6. The IMU sensor mounted on top of the sensor module measured the tilting angle while the tensile strength was being measured. The ophthalmic surgeon could refer to the tilting angle displayed on the laptop screen in real time to maintain a consistent angle while pulling the hook, thereby enhancing measurement accuracy.

## 3. Results

All calculations and statistical analyses were performed using Minitab (version 21.1.1, Minitab, State College, PA, USA). The data are presented as mean ± SD values, and the criterion for statistical significance was set as *p* < 0.05. The subjects were aged 4 to 13 years old, with a mean age of 9.4 years (range = 4–13 years), and comprised six males and five females who were scheduled for elective strabismus surgery to correct intermittent exotropia under general anesthesia. The MR and LR were compared using the Wilcoxon signed-rank test, which is designed for comparing two non-independent groups.

None of the subjects had any limitations in eye movement, and all were confirmed as physical status classification I on the American Society of Anesthesiologists system. The exclusion criteria included the presence of ocular diseases other than intermittent exotropia. Specifically, none of the included patients had nystagmus, ptosis, systemic diseases affecting extraocular muscles such as thyroid disorders, muscular or neurological conditions (e.g., myasthenia gravis), a history of ocular trauma, previous strabismus or ocular surgery, or the use of medications known to affect muscle tension (e.g., muscle relaxants).

A standardized anesthetic regimen was applied to all patients by a single anesthesiologist to produce a consistent depth of anesthesia. All patients arrived at the operating room without anticholinergic premedication. Anesthesia was induced by administering 5 mg/kg of thiopental sodium and 0.6 mg/kg of rocuronium (a nondepolarizing muscle relaxant). After performing tracheal intubation, anesthesia was maintained by administering sevoflurane with 40% oxygen in air.

**Figure 7:**
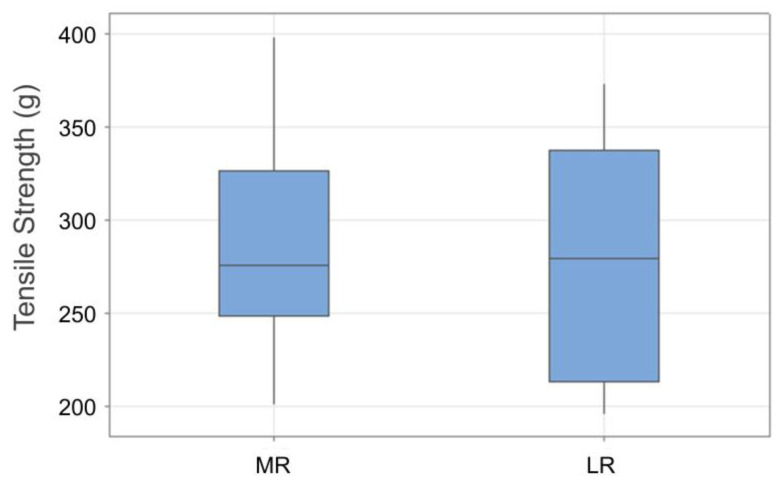
Box plots of quantitative measurement of EOM tension according to applied traction angle using the TSMD (Tension-Sensing Muscle Device). The graph shows the mean tension values (in grams) recorded during incremental traction applied to the MR and lateral rectus (LR) muscles. Each box plot shows the median, first and third quartile, and range values.

Unilateral MR resection and LR recession were performed on the nondominant eye under general anesthesia by a single surgeon (H.J.S.) in all patients. A conjunctival incision was made through the inferior nasal fornix, followed by incisions in Tenon’s capsule and the intermuscular septum. The MR was hooked, dissected, and separated from the intermuscular septum and muscle capsule (Figure 6A,B). Muscle traction was applied to the MR muscle for the same duration (10 s) and applied force (10 mm) in all cases to ensure that the muscle stimulation was as consistent as possible (Figure 6C). Similarly, the tensile force of the LR was measured using the inferior temporal approach. The measured tensile strength did not differ significantly between the MR (284.9 ± 58.3 g) and the LR (278.3 ± 64.6 g) (*p* = 0.846). Figure 7 presents the intraoperative tensile strength measurements of the MR and LR obtained from 11 patients undergoing strabismus surgery under general anesthesia. Using the TSMD, each muscle was pulled 10 mm from its insertion site with a Jameson hook, and the resulting tensile force was recorded. The measured tensile strength did not differ significantly between the MR (284.9 ± 58.3 g) and the LR (278.3 ± 64.6 g) (*p* = 0.846).

## 4. Discussion

The outcomes of strabismus surgery can vary markedly even when the same amount of EOM resection is performed. This variability arises from differences in preoperative muscle tension, which reflect underlying biomechanical properties, as well as the postoperative healing responses [21,22,23]. Traditional methods for assessing EOM tension rely on subjective evaluations made by the surgeon, which lack quantitative precision and can lead to unpredictable surgical outcomes [4,16]. We have addressed this limitation by developing a novel device that objectively measures EOM tensile strength in vivo using a strain gauge load cell integrated with a strabismus hook and a tilting sensor to increase the measurement accuracy.

The in vivo experiments performed in this study have demonstrated that our novel device provides reliable and reproducible measurements of EOM tensile strengths in anesthetized patients undergoing strabismus surgery. We found that the mean tensile strengths of the MR and LR were 284 and 278 g, respectively. There are previous reports of passive tension values ranging from 45.6 to 74.8 g for the MR and from 48.3 to 59.1 g for the LR [4,19] when assessed using forceps, while reported active tension values measured during voluntary eye movements have ranged between 88 and 102 g [16]. To the best of our knowledge, no previous studies have measured the EOM tensile strength using a methodology similar to that applied in the present study, and so direct comparisons with existing data are not feasible. However, our measurements exceeded previously reported passive and active tension values. These discrepancies were likely due to our methodology involving pulling the muscle in opposite directions, which will result in higher tensile force values. The measured force is highly dependent on the degree of muscle stretch, and in our study, we standardized the stretch distance at 10 mm to ensure maximal muscle tautness—the measured values would have been lower if a shorter stretch distance had been applied. To optimize the clinical applicability of our device, future research should determine the ideal stretch distance that best reflects the biomechanical properties of EOMs.

Various quantitative methods have been developed to measure EOM tension, but each has limitations in clinical use. Implantable force transducers offer precise force measurements but require surgical insertion and are mainly restricted to animal or laboratory settings due to their invasiveness [20,24]. Dial tension gauges involve tendon anchoring and are influenced by the operator technique and hook angle used, limiting reproducibility and real-time feedback [25]. Intraoperative strain gauge systems provide accurate data but require complex calibration and bulky equipment, making them impractical for routine orbital surgery.

Some devices such as strain gauge systems allow comparisons of contractile properties during strabismus surgery, but they remain impractical for routine use due to their complex setup and their variability with the surgical settings [26]. Similarly, real-time measurements of muscle forces and orbital tissue stiffness using length-tension forceps, combined with ultrasonic eye positioning, provide more accurate results, but such approaches require trained personnel and specialized instrumentation [27]. The method proposed in the present study offers significant advantages over these previous methods. Replacing the conventional strabismus hook with a sensor-equipped hook allows for real-time intraoperative measurements of EOM tensile strength while pulling the muscle in opposite directions, without requiring additional incisions, procedural steps, or modifications to the surgical workflow. This noninvasive and user-friendly approach seamlessly integrates into standard strabismus surgical procedures, making it a practical tool for both clinical applications and research into ocular motility disorders.

Recently, implantable strain sensors such as the wireless suture-integrated system reported by Yang et al. [28] have demonstrated the real-time in vivo monitoring of ligament strain using double-helix PEDOT/PSS-coated sutures and inductive coupling circuits. These systems achieve high precision and biocompatibility, supporting long-term implantation and strain detection at thresholds as low as 0.25% with wireless readout via resonant frequency shifts. However, these systems require complex fabrication, precise coil alignment, and surgical implantation, which limit their suitability for short-duration intraoperative procedures like strabismus surgery. In contrast, our TSMD does not require implantation or preoperative setup. It provides instantaneous quantitative force measurement using a strain gauge-equipped hook and is fully compatible with standard extraocular muscle exposure techniques. While the absolute strain resolution is lower, our approach offers significantly greater surgical practicality, particularly for ophthalmologists who operate in small anatomical spaces and require immediate feedback during muscle manipulation.

This method of bidirectional tension application may more accurately simulate the true intraoperative mechanical resistance experienced during muscle manipulation compared to conventional forceps-based or passive measurement techniques. By providing real-time, quantitative data, the TSMD allows the surgeon to assess EOM elasticity and resistance during the procedure, which may help to reduce surgical subjectivity and enhance the predictability of outcomes. These objective measurements can also support personalized surgical planning by enabling adjustments to resection or recession amounts based on preoperative tension characteristics. Furthermore, the TSMD may assist in distinguishing between paretic, restrictive, and normal muscle conditions intraoperatively, particularly in complex or ambiguous cases such as those involving thyroid eye disease or muscle fibrosis.

Recent advances in organic electronic biosensors underscore the value of lightweight, flexible, and low-power systems for real-time monitoring. OFETs and OECTs have been used in glucose, DNA, ion, and gas sensing, with OECTs offering enhanced sensitivity and selectivity due to their electrolyte-modulated conductivity [29]. Moreover, AI-based signal prediction in ultrafast hydrogen sensors has enabled sub-second detection using early-stage data, improving both speed and accuracy [30]. These innovations suggest promising directions for improving the TSMD through faster response, smart signal processing, and the integration of flexible sensor systems, supporting more precise and responsive intraoperative measurements.

Despite its considerable potential, this study has several limitations that should be acknowledged. The accurate measurement of tensile strength ideally requires the complete removal of elastic connective tissues surrounding the EOMs. However, achieving consistent dissection across all cases is challenging in clinical practice, which can affect measurement reliability. Additionally, this study provides only a preliminary validation of the proposed method for quantifying EOM tensile strength. Since no previous research has evaluated EOM tension using this approach, larger-scale studies are needed to assess its clinical significance. Future investigations could examine whether preoperative tensile strength measurements correlate with postoperative outcomes, such as the risk of overcorrection or undercorrection. For instance, higher intraoperative tension values observed during resection may be associated with greater degrees of surgical correction. The findings from such studies could help to refine surgical decision-making and improve the predictability of strabismus surgery.

## 5. Conclusions

In this study, we developed a novel device for quantitatively measuring the preoperative EOM tensile strength, which could aid strabismus surgery planning. By providing objective real-time measurements, this method may help to explain individual differences in muscle elasticity, thickness, and ultrastructural variations, potentially allowing for more personalized surgical approaches. Assessing the mechanical properties of EOMs in this way could contribute to improved surgical predictability and patient outcomes. Given its compact design and ease of integration into clinical practice, the new device may be suitable for applications in both research and clinical settings. Future studies could further explore the potential role of the device in characterizing EOM properties in restrictive strabismus, myopathies, and other ocular motility disorders.

## Data Availability

The data presented in this study are available upon request from the corresponding author due to restrictions imposed by the Institutional Review Board, which approved the study protocol.
